# Validation of the German version of the pregnancy-related anxiety scale (PrAS): psychometric properties across all trimesters of pregnancy

**DOI:** 10.1186/s12884-023-05787-z

**Published:** 2023-06-24

**Authors:** Tobias Weigl, Robyn Brunton, Rachel Dryer, Susan Garthus-Niegel

**Affiliations:** 1 Psychology School, Fresenius University of Applied Sciences Düsseldorf, Düsseldorf, Germany; 2 Charles Sturt University, Bathurst, NSW 2795 Australia; 3 Australian Catholic University, Strathfield, NSW 2135 Australia; 4 Institute for Systems Medicine (ISM), Faculty of Medicine, Medical School Hamburg, Hamburg, Germany; 5 Department of Childhood and Families, Norwegian Institute of Public Health, Oslo, Norway; 6 Institute and Policlinic of Occupational and Social Medicine, Faculty of Medicine, Technische Universität Dresden, Dresden, Germany

**Keywords:** Pregnancy, Anxiety, Screening, Psychometric properties, Questionnaire

## Abstract

**Background:**

Pregnancy-related anxiety has received greater research attention recently given its association with adverse outcomes (e.g., negative birth experiences). The Pregnancy-related Anxiety Scale (PrAS) offers the possibility to assess pregnancy-related anxiety, but no German version is available. Therefore, the aim of this study was to validate a German version of the PrAS, a comprehensive measure with eight dimensions.

**Methods:**

Pregnant women of any parity or gestation completed an online survey consisting of the PrAS, PRAQ-R2, and measures of anxiety, depression, and resilience. The PrAS was translated into German (PrAS-G) using the back-translation method. Data were subjected to confirmatory factor analysis and inferential statistics.

**Results:**

Complete data were provided by 443 women. Participants were predominantly German nationals, partnered, and well-educated with a planned pregnancy. Approximately half were nulliparous. The eight-factor model was well fitting and consistent with the development of the original PrAS. Criterion-related validity was demonstrated by strong correlations with similar measures (PRAQ-R2, anxiety, and depression) and lower correlations with resilience scores. Predictive validity was shown by group comparisons for: planned versus unplanned pregnancy, trimester, and parity.

**Conclusions:**

The PrAS-G provides a broader assessment of pregnancy-related anxiety than existing measures. Initial evaluation has demonstrated convergent, divergent, and predictive validity, excellent internal consistency, and good model fit indicating promising psychometric properties. The PrAS-G offers a comprehensive assessment of pregnancy-related anxiety which will enable tailored interventions aiming to improve birth experience and well-being of expectant mothers.

## Introduction

Being pregnant and having a child are commonly perceived as joyful experiences. However, pregnancy can also be accompanied by a decline in mental health and women might suffer from elevated levels of symptoms of depression and anxiety [1]. While it was possible to establish a relatively clear outline of prenatal depression, this has been somewhat difficult for prenatal anxiety. In previous years, it has been unclear if pregnancy-related anxiety (PrA) can be distinguished from general anxiety. In fact, results suggest that PrA needs to be seen as a unique set of symptoms [2]. The concept of PrA includes dimensions like fear of childbirth, body image, loss of fetus, worries that the baby might die or get injured, financial and family support among others [3].

PrA has received a greater research focus in the last 10–15 years [4]. Several studies point to adverse effects on women´s mental health such as negative birth experiences and birth trauma due to PrA [5–10]. Further studies even suggest additional detrimental consequences for the offspring associated with PrA, like preterm birth, low birth weight, or development of difficult infant temperament [2, 3, 11–16]. So far, PrA has not been included in the Diagnostic and Statistical Manual of Mental Disorders 5 (DSM-5) or the International Classification of Diseases 11 (ICD-11) as a diagnostic entity. Nevertheless, the prevalence of this anxiety may be as high as 11% with rates varying depending on the country and parity [17]. Other influencing factors include the trimester at assessment and if the pregnancy was planned [18, 19].

Since PrA has not been conceptualized unequivocally so far, fears which may occur during pregnancy were not adequately assessed [3, 6]. As a consequence, there seems to be an ongoing controversy regarding the scales used to identify PrA. Systematic reviews have identified seven scales specifically designed for the assessment of PrA in English speaking countries, which offer additional properties and higher validity when compared to general measures of anxiety. However, reviews point to the need for the development of a scale for PrA that has sound psychometric properties [20, 21].

Currently, only The Pregnancy-Related Anxiety Questionnaire-Revised 2 (PRAQ-R2) has been validated in German with low to medium levels of PrA, as in the original version [22, 23]. Due to missing cutoff values, prevalence for Germany are not available. This scale is considered a useful tool due to its brevity. However, the PRAQ-R2 only covers three relevant features of PrA: childbirth, baby concerns, and appearance-related concerns. The Pregnancy-related Anxiety Scale (PrAS) was therefore developed consistent with the objective of both covering relevant aspects of PrA as well as ensuring high psychometric quality [24, 25]. The PrAS provides a more comprehensive assessment of PrA, assessing eight facets (i.e., childbirth, baby concerns, appearance, attitudes towards medical staff, acceptance, avoidance, attitudes towards childbirth, and worry about self) and offers the possibility to identify particular facets of PrA, beyond the assessment provided by the PRAQ-R2. This might help to provide targeted interventions that are adapted to the specific needs of women during pregnancy, which has profound individual clinical relevance. We therefore aim to translate the PrAS to German and validate its psychometric properties.

## Method

### Participants and procedure

For this cross-sectional study, pregnant women in all trimesters were recruited using a multi-faceted approach. First, recruitment took place via Facebook and Instagram pages focusing on pregnancy in any way. Second, women were recruited using posters and flyers at local gynecological offices, enabling access to the online study by a QR-code. To encourage completion of the questionnaire, an incentive was offered (a chance to win one out of ten €15 gift cards). The survey was conducted online with the use of the platform EFS by QuestBack (www.unipark.de). After providing informed consent online, participants completed the survey on a smartphone or computer in an environment of their choice. Women were informed about their right to withdraw from the study at any given time. Additionally, they were advised to seek professional help if needed and numbers were provided to support services (i.e., German National Suicide and Crisis Line). The survey took approximately 15 min to complete. Nulliparous and multiparous women in all trimesters of pregnancy were included. Since we intended to collect data from a community sample, participants with mental disorders were also included. Exclusion criteria were younger age than 18 years and insufficient German language skills. The study protocol was approved by the Ethics Review Board of the University of Dresden (approval no. SR-EK-282,062,020) and the study was performed in accordance with the Declaration of Helsinki. All data were stored anonymously and in accordance with the German General Data Protection Regulation. Originally, 572 women took part in the study. However, after exclusion due to incomplete data, the sample consisted of 443 pregnant women. As previously suggested, not less than ten cases per indicator variable represent an acceptable sample size for CFA. With a total sample of N = 443, this prerequisite was fulfilled [26].

### Measures

#### Sociodemographic and obstetric characteristics

Sociodemographic information included maternal age, relationship status, educational status, and nationality. Questions regarding obstetric characteristics assessed planned vs. unplanned pregnancy (assessed with the item ‘Did you plan to get pregnant?’ – ‘Yes’ vs. ’No’), trimester, and parity.

### Psychological scales

#### Pregnancy-related Anxiety Scale (PrAS)

The Pregnancy-related Anxiety Scale (PrAS) provides an assessment of maternal pregnancy-related anxiety [24, 27]. The PrAS consists of 32 items rated on a four-point-scale from 1 (not at all) to 4 (very often) with 6 reverse-scored items. The PrAS has eight factors: Childbirth Concerns (6 items), Body Image Concerns (5 items), Attitudes Towards Childbirth (3 items), Worry About Self (6 items), Baby Concerns (3 items), Acceptance of Pregnancy (3 items), Avoidance (3 items), and Attitudes Towards Medical Staff (3 items). Higher values indicate greater anxiety. The PrAS has excellent internal consistency reliability with α = 0.92 for the total scale and ≥ 0.80 for all subscales [24]. In the current study α = 0.90 for the total scale with all subscales ≥ 0.76. McDonald’s ω was 0.89. The scale has demonstrated convergent and divergent validity [25, 28]. The PrAS was translated into German, using the back-translation method [29]. All translations were done by a professional translation service and different independent translators were responsible for the translation from English to German and from German to English. The original authors were consulted for an assessment of the back-translated version in English, which resulted in minimal changes to the wording in the German version. During a pretest, twenty pregnant women who were not familiar with the aims of our study reported no difficulties regarding the wording when filling in the items of the German version of the PrAS.

#### Pregnancy-Related Anxiety Questionnaire-Revised 2 (PRAQ-R2)

The Pregnancy-Related Anxiety Questionnaire-Revised 2 (PRAQ-R2) is an advancement from the Pregnancy-Related Anxiety Questionnaire, specifically designed to assess anxiety experienced by women during pregnancy [22, 23, 30, 31]. It consists of ten items rated on a five-point scale from 1 (absolutely not relevant) to 5 (very relevant). Sum scores can be calculated for the subscales Fear of Giving Birth (3 items with a range from 3 to 15), Worries of Bearing a Physically or Mentally Handicapped Child (4 items with a range from 4 to 20), Concerns about own Appearance (3 items with a range from 3 to 15), as well as the total scale (range from 10 to 50). The German version of the PRAQ-R2 was used and the internal consistency for the current study was α = 0.85 and ω = 0.82 respectively.

#### Edinburgh Postnatal Depression Scale (EPDS)

Symptoms of depression were measured with the German version of the Edinburgh Postnatal Depression Scale (EPDS). The EPDS is a commonly used self-report scale to assess depression in the postpartum period, but has also been validated for antenatal use [30, 32–36]. The EPDS consists of 10 items rated on a four-point scale from 0 to 3 with varying response options. The sum score ranges from 0 to 30. Higher scores reflect higher levels of depression. The internal consistency for the current study was α = 0.88 and ω = 0.88 respectively.

#### Depression, Anxiety and Stress Scale (DASS-21)

The Depression, Anxiety and Stress Scale-21 (DASS-21) consists of 21 items with three subscales assessing symptoms of depression, anxiety, and stress. The DASS-21 has been validated in postpartum mothers [37, 38]. Items are rated on a scale from 0 (did not apply to me at all) to 3 (applies to me very much or most of the time), and sum scores can be calculated for each scale. In the present study, the German version of the subscale “DASS-Anxiety” was used [30, 39]. The internal consistency for the current study was α = 0.81 and ω = 0.82 respectively.

#### Brief Resilience Scale (BRS)

The Brief Resilience Scale (BRS) is a self-report scale measuring how well a person is able to recover after experiencing stressful events, which is defined as resilience by the original authors (Smith et al., 2008). The scale consists of 6 items and rated on a five-point-scale from 1 (strongly disagree) to 5 (strongly agree). Items 2, 4, and 6 are reverse-scored, since they are negatively phrased [40, 41]. Higher scores indicate a greater ability to bounce back from stress. The German version shows a unidimensional structure with good internal consistency reliability, α = 0.85 in the original validation study. The internal consistency for the current study was α = 0.79 and ω = 0.78 respectively.

### Statistical analysis

Reliability was estimated with Cronbach’s α using IBM SPSS statistics version 28 for windows. Additionally, internal consistency was also estimated with McDonald’s ω, which is a less biased estimate than Cronbach’s α to provide extensive comparability [42]. Confirmatory factor analysis (CFA) was performed using IBM SPSS Amos version 28. Fit indices included Root Mean Square Error of Approximation (RMSEA), Standardized Root Mean Square Residual (SRMR), Comparative Fit Index (CFI), and Tucker-Lewis Index (TLI). Good (and adequate, respectively) model fit is indicated by RMSEA ≤ 0.06 (0.06–0.08), SRMR ≤ 0.05 (0.05–0.08), and CFI, as well as TLI ≥ 0.95 (0.90–0.95) [43]. IBM SPSS statistics version 28 for windows was used to yield correlation coefficients for convergent and divergent validity and conduct secondary analyses with tests for group differences. To test for significant differences between women with planned versus unplanned pregnancies, different trimesters, and parity, Welch´s t-test and Welch´s ANOVA were used, since some of the subscales of the PrAS showed no homogeneity of variance. Post-hoc comparisons were computed with the Games-Howell-Test [44, 45].

## Results

### Sample characteristics

The final sample included *N* = 443 women (*M*_age_ = 31.9, *SD* = 4.2). Over 90% of women were German nationals, with most being married or cohabitating. The women in this sample were well educated with roughly 81% having a general qualification for university entrance. More than 86% stated that the current pregnancy was planned and approximately half of women were expecting their first child. Table 1 provides full details.

**Table 1:** Sociodemographic and obstetric characteristics of sample

Variables	(n = 443)	
*Age (years)* ^*1*^	31.9 ± 4.17 (20–43)	
*Relationship status (n / %)*		
Engaged / married	300 / 67.7	
Cohabitating	133 / 30.0	
Divorced / living apart	10 / 2.3	
*Education (n / %)*		
No degree	0 / 0	
Secondary education	84 / 19.0	
General qualification for university entrance	359 / 81.0	
University degree	217 / 48.9	
*Nationality (n / %)*		
German	420 (94.8)	
Other	23 (5.2)	
*Planned pregnancy (n / %)*		
Yes	383 (86.5)	
No	60 (13.5)	
*Trimester* ^*1*^		
All trimesters	26.33 ± 10.30 (1–43)	
1st trimester (≤ 13 weeks, n = 56)	7.93 ± 2.74 (1–12)	
2nd trimester (13- ≤ 26 weeks, n = 122)	20.48 ± 3.76 (13–26)	
3rd trimester (≥ 26 weeks, n = 265)	34.75 ± 3.95 (27–43)	
*Parity (n / %)*		
Nulliparous (expecting first child)	227 (51.2)	
Primiparous	147 (33.2)	
Multiparous	69 (15.6)	

^**1**^ M ± SD / (range).

### Reliability

Analysis of the internal consistency by Cronbach’s α resulted in excellent reliability of 0.90 and a Cronbach´s α ≥ 0.76 for all eight subscales. Further analyses reached a McDonald’s ω = 0.89 for the total scale. Thus, both measures indicate sound reliability [46].

### Confirmatory factor analysis (CFA)

**Fig. 1:**
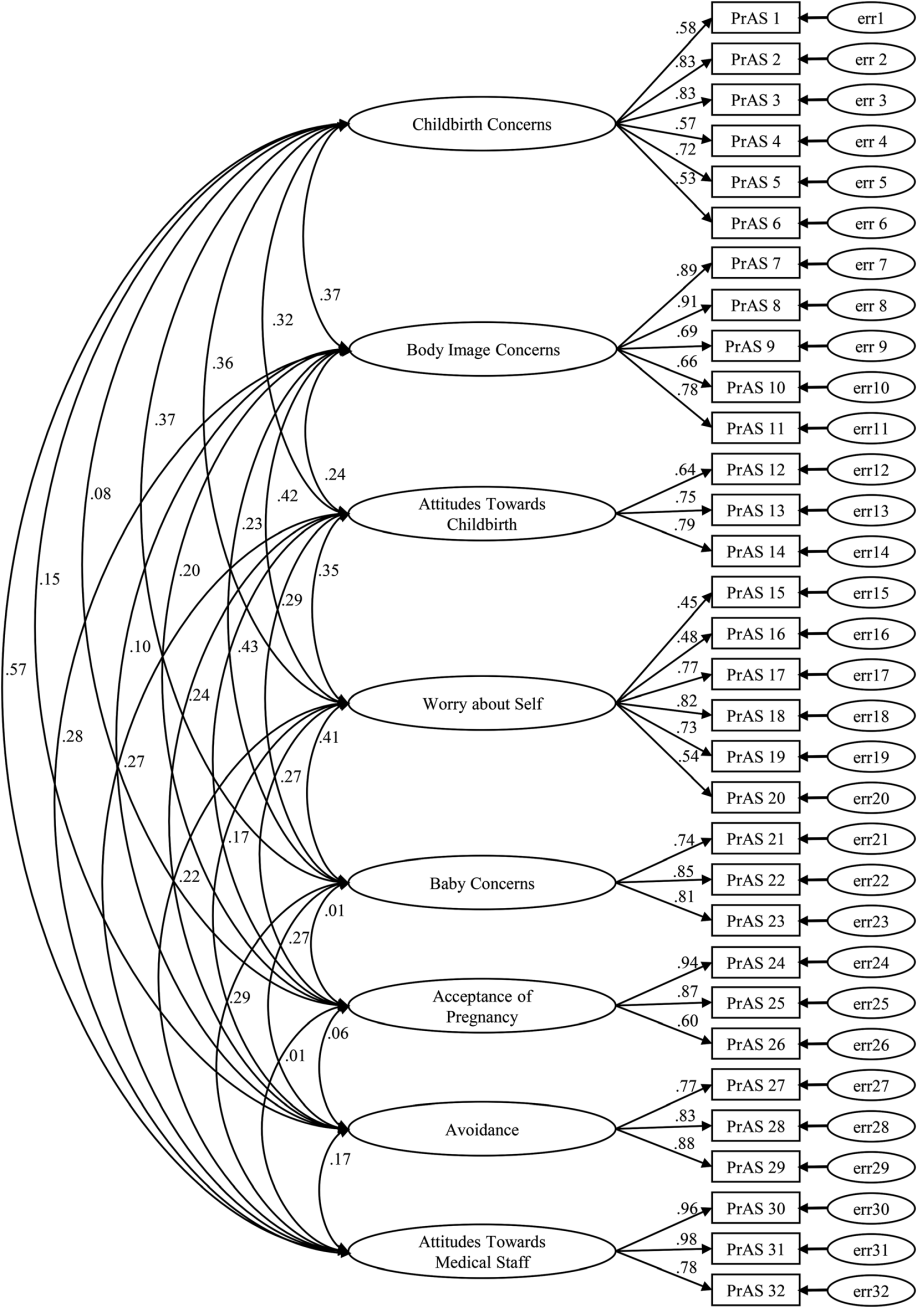
Standardized factor loadings of Model 1 with eight factors. Correlations between factors are also indicated

To confirm the underlying factor structure of the translated scale, data were subjected to a theory-based CFA. For the original English version of the PrAS, two similar models showed adequate fit in previous validation studies and were tested in the current sample [24, 25]. The Kaiser-Meyer-Olkin measure of sampling adequacy test resulted in a coefficient of 0.85 and Bartlett’s Test of Sphericity was significant (χ^2^(496) = 8146.65, *p* < .001). Both results confirmed data suitability in accordance with previous guidelines [47, 48]. Initially, we tested a model with eight factors (Model 1). Those factors included: Childbirth Concerns, Body Image Concerns, Attitudes Towards Childbirth, Worry About Self, Baby Concerns, Acceptance of Pregnancy, Avoidance, Attitudes Towards Medical Staff. Model 1 (χ^2^(436) = 1262.57, χ^2^/df = 2.896) yielded a good fit to the data evidenced by the fit indices RMSEA = 0.065, SRMR = 0.069, CFI = 0.895, and TLI = 0.880. We also tested a model with nine factors (Model 2) in which items 15 and 16 were detached from the scale Worry About Self, building an additional scale Worry About Motherhood. Model 2 (χ^2^(428) = 1094.305, χ^2^/df = 2.557) was a slightly better fit to the data, with RMSEA = 0.059, SRMR = 0.062, CFI = 0.915, TLI = 0.902. Since both analyses resulted in similar results, we proceeded with the eight-factor model (Model 1) as proposed for the original version of the PrAS [24]. This way, the comparability of the English and German version of the PrAS can be ensured. Standardized factor loadings of Model 1 are shown in Fig. 1.

### Convergent and divergent validity

To test criterion-related validity of the PrAS with other measures, correlations between the PrAS and its subscales and convergent (i.e., PRAQ-R2 and its subscales, EPDS, and DASS-Anxiety subscale) and a divergent measure (BRS) were calculated. As expected, the PrAS and the PRAQ-R2 showed strong linear relationships between the scales overall and the related subscales. For example, the PrAS subscales Baby Concerns and Body Image Concerns were strongly correlated with the PRAQ-R2 subscales Worries of Bearing a Physically or Mentally Handicapped Child and Concerns about Own Appearance, respectively. There were moderate correlations between the PrAS subscales Childbirth Concerns and Attitudes about Childbirth and the PRAQ-R2 Fear of Giving Birth as well as for the PrAS with the EPDS and the DASS-Anxiety. The divergent correlation of the PrAS sum score and almost all subscales (except Avoidance) with the BRS resulted in a weak negative relationship. See Table 2 for further details.

**Table 2:** Correlations of the PrAS total scale and subscales with convergent and divergent measures (n = 443)

	*M*	*(SD)*	PrAS total scale	Childbirth Concerns	Body Image Concerns	Attitudes Towards Childbirth	Worry About Self	Baby Concerns	Acceptance of Pregnancy	Avoidance	Attitudes Towards Medical Staff
PRAQ-R2 total scale	22.03	(7.09)	0.780^***^	0.552^***^	0.627^***^	0.451^***^	0.475^***^	0.626^***^	0.189^***^	0.332^***^	0.373^***^
PRAQ-R2 FoGB	7.23	(2.75)	0.651^***^	0.575^***^	0.368^***^	0.560^***^	0.337^***^	0.358^***^	0.150^***^	0.317^***^	0.368^***^
PRAQ-R2 WaHC	8.36	(3.44)	0.526^***^	0.341^***^	0.251^***^	0.290^***^	0.387^***^	0.763^***^	0.061	0.275^***^	0.248^***^
PRAQ-R2 CoA	6.44	(3.13)	0.619^***^	0.373^***^	0.824^***^	0.212^***^	0.357^***^	0.268^***^	0.231^***^	0.173^***^	0.252^***^
DASS-Anxiety	3.90	(4.04)	0.554^***^	0.336^***^	0.364^***^	0.169^***^	0.646^***^	0.289^***^	0.171^***^	0.250^***^	0.303^***^
EPDS	9.29	(5.89)	0.587^***^	0.302^***^	0.435^***^	0.254^***^	0.721^***^	0.302^***^	0.285^***^	0.112^*^	0.271^***^
BRS	3.35	(0.70)	-0.364^***^	-0.208^***^	-0.192^***^	-0.269^***^	-0.399^***^	-0.204^***^	-0.182^***^	-0.068	-0.195^***^

^***^p < .001; ^**^p < .01; ^*^p < .05.

Instruments: *PrAS* Pregnancy-related Anxiety Scale;

*PRAQ-R2* Pregnancy-Related Anxiety Questionnaire-Revised 2;

*PRAQ-R2-FoGB* Pregnancy-Related Anxiety Questionnaire-Revised 2, subscale Fear of Giving Birth;

*PRAQ-R2-WaHC* Pregnancy-Related Anxiety Questionnaire-Revised 2, subscale Worries of Bearing a Physically or Mentally Handicapped child; *PRAQ-R2-CoA* Pregnancy-Related Anxiety Questionnaire-Revised 2, subscale Concerns about own Appearance;

*DASS-Anxiety* Depression, Anxiety and Stress Scale, subscale Anxiety;

*EPDS* Edinburgh Postnatal Depression Scale;

*BRS* Brief Resilience Scale.

### Group comparisons for planned pregnancy, trimester, and parity

The sum scores of the total scale as well as the subscales of the PrAS were tested for significant differences with regard to planned pregnancy, trimester, and parity.

#### Planned pregnancy

Compared to women with a planned pregnancy (*N* = 383), women whose pregnancy was unplanned (*N* = 60) had higher scores for the PrAS and all subscales. Welch´s test confirmed that there were significant differences for planned versus unplanned pregnancy for the PrAS sum score as well as the subscales Childbirth Concerns, Body Image Concerns, Attitudes Towards Childbirth, Worry About Self, and Acceptance of Pregnancy. No statistically significant differences were found for Baby Concerns, Avoidance, or Attitudes Towards Medical Staff (all *p* > .05). Table 3 provides further details including the mean scores for each group.

**Table 3:** Mean (SD) values of PrAS total and subscale sums, and differences between women whose pregnancies were either planned or unplanned

	*Planned pregnancy (n = 383)*	*Unplanned pregnancy* *(n = 60)*	
	*M (SD)*	*M (SD)*	*p-value*
PrAS total scale Childbirth Concerns Body Image Concerns Attitudes Towards Childbirth Worry about Self Baby Concerns Acceptance of Pregnancy Avoidance Attitudes Towards Medical Staff	54.30 (12.14) 11.25 (3.89) 8.68 (3.42) 6.41 (2.14) 9.60 (2.98) 4.90 (1.82) 3.62 (1.38) 4.07 (1.92) 5.76 (2.57)	64.40 (14.17) 12.75 (4.65) 11.68 (4.38) 7.07 (2.14) 11.25 (2.14) 5.27 (1.83) 6.18 (2.21) 4.08 (1.82) 6.12 (2.62)	< 0.001^***^ 0.01^*^ < 0.001^***^ 0.015^*^ < 0.001^***^ 0.078 < 0.001^***^ 0.476 0.164

^***^p < .001; ^**^p < .01; ^*^p < .05;

#### Trimester

**Fig. 2:**
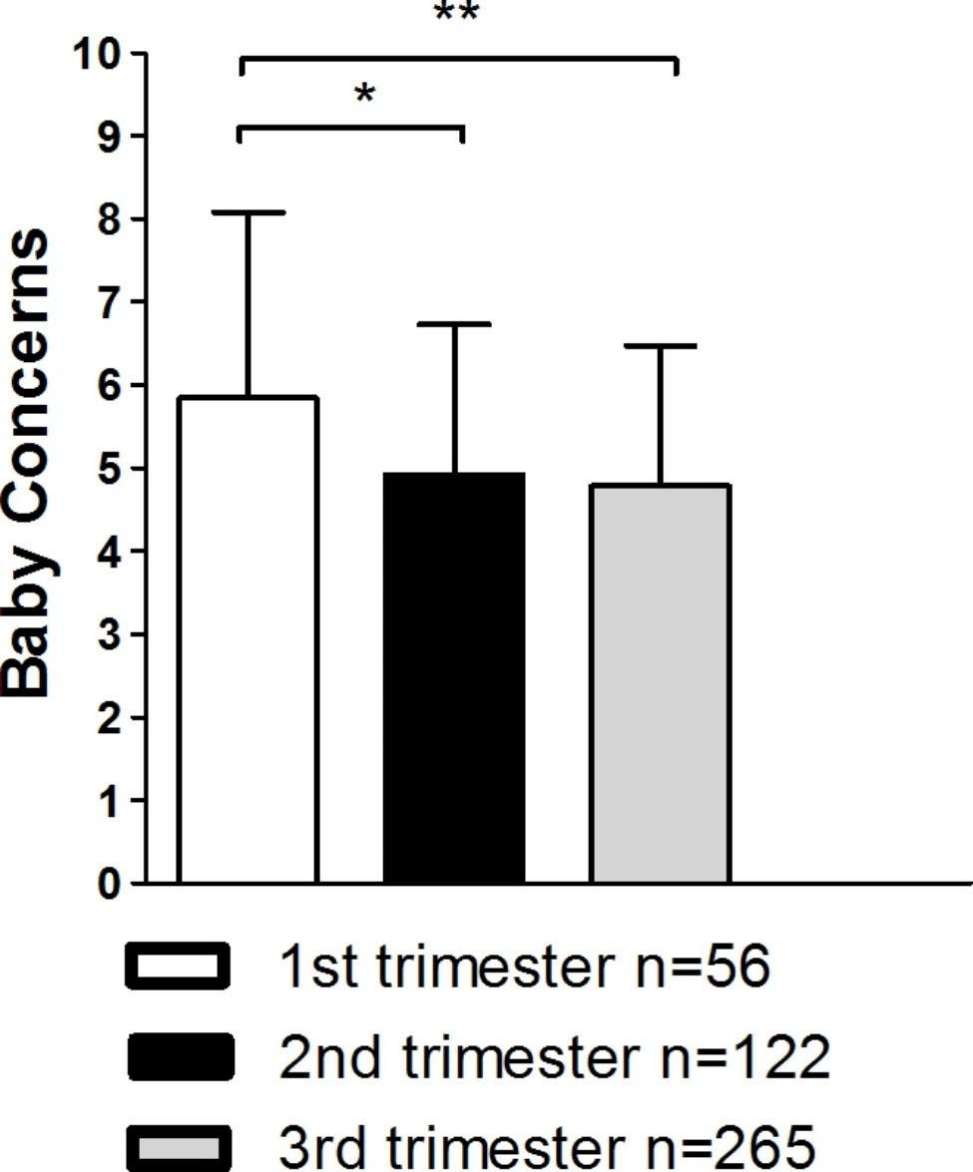
Scores of the scale Baby Concerns for all trimesters; *** *p* < .001; ** *p* < .01; * *p* < .05; Data are shown as mean values ± SD.

When comparing PrAS scores of women in different trimesters, Welch´s ANOVA revealed significant differences for the subscales Attitudes Towards Childbirth *F*_(2,147.42)_ = 3.11; *p* < .05 and Baby Concerns *F*_(2,141.63)_ = 5.43; *p* < .01. Nevertheless, in Games-Howell post-analyses significant differences could only be found for the scale Baby Concerns. Women in the first trimester had significantly higher scores than women in the second (*p <* .05; 0.95, 95%-CI[0.16, 1.74]) or third trimester (*p* < .01; 1.06, 95%-CI[0.29, 1.83]). See Fig. 2 for further details.

#### Parity

**Fig. 3:**
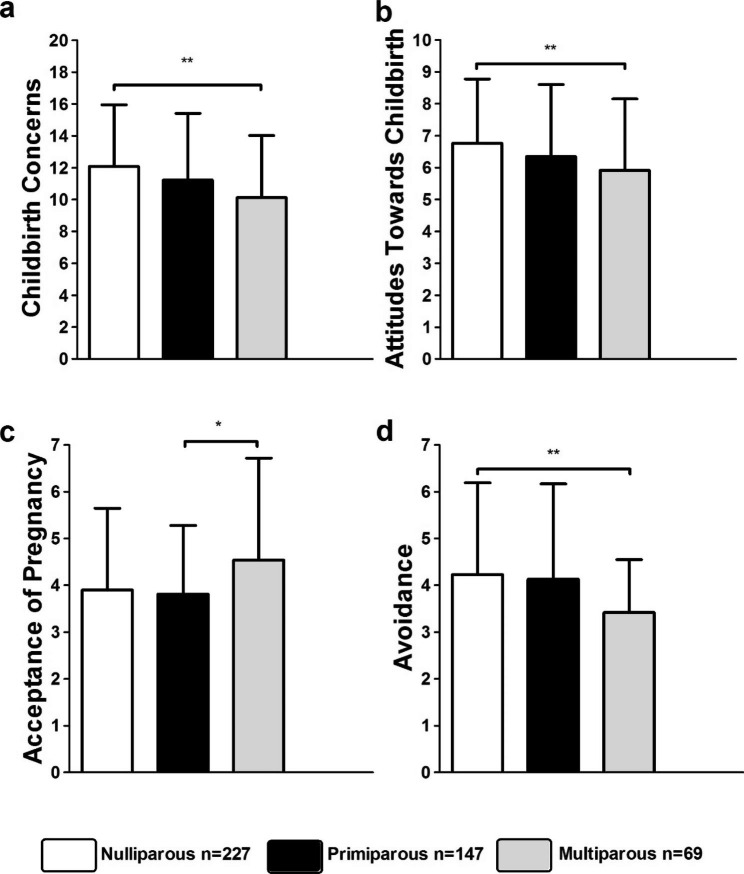
Scores of the scale Childbirth Concerns (a), Attitudes Towards Childbirth (b), Acceptance of Pregnancy (c) and Avoidance (d) for nulliparous, primiparous and multiparous women; *** *p* < .001; ** *p* < .01; * *p* < .05; Data are shown as mean values ± SD.

We also tested for differences between women who were either nulliparous, primiparous, or multiparous. Using Welch´s ANOVA, significant differences emerged for the subscales Childbirth Concerns (*p <* .05), Attitudes Towards Childbirth (*p* < .05), Acceptance of Pregnancy (*p* < .05), and Avoidance (*p* < .001). Games-Howell post-hoc analyses revealed significant differences between PrAS scores of nulliparous and multiparous women for the scales Childbirth Concerns (*p* < .01; 1.88, 95%-CI[0.61, 3.15]), Attitudes Towards Childbirth (*p <* .05; 0.83, 95%-CI[0.12, 1.54]), and Avoidance (*p* < .001; 0.81, 95%-CI[0.36, 1.25]). Further, significant differences emerged between scores of primiparous and multiparous women for the subscale Acceptance of Pregnancy (*p* < .05; − 0.73, 95%-CI[-1.41, − 0.04]). Table 4; Fig. 3 provide the full details.

**Table 4:** Mean (SD) values and differences between nulliparous, primiparous and multiparous women in the PrAS total scale and the subscales (n = 443)

	PrAS total scale	Childbirth Concerns	Body Image Concerns	Attitudes Towards Childbirth	Worry About Self	Baby Concerns	Acceptance of Pregnancy	Avoidance	Attitudes Towards Medical Staff
*Parity*	*M (SD)*	*M (SD)*	*M (SD)*	*M (SD)*	*M (SD)*	*M (SD)*	*M (SD)*	*M (SD)*	*M (SD)*
Nulliparous (n = 227)	56.77 (12.71)	12.08 (3.87)	9.05 (3.71)	6.76 (2.02)	9.77 (3.01)	5.09 (1.83)	3.90 (1.75)	4.23 (1.96)	5.96 (2.51)
Primiparous (n = 147)	55.35 (13.40)	11.22 (4.19)	9.17 (3.75)	6.35 (2.25)	10.05 (3.36)	4.87 (1.91)	3.81 (1.47)	4.13 (2.04)	5.74 (2.78)
Multiparous (n = 69)	52.72 (12.02)	10.13 (3.89)	9.06 (3.89)	5.92 (2.23)	9.54 (2.75)	4.67 (1.62)	4.54 (2.18)	3.42 (1.13)	5.45 (2.34)
	*F*_(2,186.31)_ = 2.95; *p* = .055	* F*_(2,182,23)_ = 6.53; *p* = .002	* F*_(2,184.45)_ = 0.05; *p* = .951	* F*_(2,176.62)_ = 4.44; *p* = .013	* F*_(2,188.56)_ = 0.49; *p* = .486	* F*_(2,190.68)_ = 1.86; *p* = .159	* F*_(2,171.75)_ = 3.18; *p* = .044	* F*_(2,231.52)_ = 10.30; *p* < .001	* F*_(2,187.11)_ = 1.27; *p* = .285

## Discussion

The main purpose of this study was to establish a German version of the PrAS and to examine its psychometric properties. The German version of the PrAS (PrAS-G) provides a more comprehensive assessment of PrA than the German version of the PRAQ-R2 [3, 20, 22, 25].

Using back-translation and a pretest, the PrAS-G was applicable for data acquisition and further analysis. Both models tested in CFA showed a good fit to the data. Despite the fact that in our CFA a solution with nine factors showed a slightly better fit to the data, we proceeded with the eight-factorial solution in accordance with the original version. This way, comparability across different language versions can be enabled. Due to the fact that results of CFA are dependent on the sample, further studies are needed in diverse cohorts to optimize analyses of the factorial structure. As expected, the subscales Baby Concerns and Body Image Concerns of the PrAS correlated with the subscales PRAQ-R2-Worries of Bearing a Physically or Mentally Handicapped child, PRAQ-R2-Concerns about own Appearance and the subscales Childbirth Concerns and Attitudes about Childbirth of the PrAS correlated with the PRAQ-R2-Fear of Giving Birth. The constructs measured in these scales are obviously similar. However, as has been stated before, further aspects of PrA are covered by the PrAS-G. For example, the PrAS-G includes scales on speculations about behavior of medical staff. This enhanced concept helps to identify PrA in women in more detail, providing a much better understanding of anxiety related to pregnancy. Correlations of the PrAS with the DASS-Anxiety were moderate and reflect the fact that the PrAS-G also measures general anxiety symptoms in addition to specific concerns. When it comes to divergent validity, the PrAS sum score and most of the subscales were negatively correlated with the BRS. Thus, both scales are measuring conceptually different constructs which accounts for divergent validity.

Several group comparisons helped to confirm the ability of the PrAS and its subscales to differentiate between women from different populations. Particularly, we compared women in our sample with planned versus unplanned pregnancies. In accordance with previous findings, women who were pregnant without intention seem to experience more PrA. Reasons for this are manifold and include a lack of information on pregnancy and birth, no steady relationship, deficient preparation, and general refusal to have a child [49, 50]. This is reflected by higher scores on the PrAS sum score as well as the subscales Childbirth Concerns, Body Image Concerns, Attitudes Towards Childbirth, Worry About Self, and Acceptance of Pregnancy. In contrast, scores of the subscales Baby Concerns, Avoidance, and Attitudes Towards Medical Staff did not differ between groups. This implies, that women who were pregnant without intention, seem to have similar concerns regarding the health of their baby, deciding which way of delivery might be the best, and how hospital staff will interact with them during their hospital stay. This overlap can be explained by the fact that several aspects of PrA are equally important for pregnant women, regardless of whether the pregnancy was planned or not. In particular, aspects of pregnancy and birth which are only partially predictable or controllable seem to be highly relevant for all (becoming) mothers.

With regard to trimesters, significant differences emerged for the subscale Baby Concerns exclusively. Women in the second and third trimester seem to worry less about the physical and mental health of their baby than women in the first trimester. Previous studies have shown that the first trimester poses a time of high uncertainty and ambiguity which subsides over the course of pregnancy [51, 52]. In particular, the predictability of a positive outcome of pregnancy for both woman and child, the ability to cope with potential adversities, and the adaptation to altered circumstances improve [53].

Scores of other subscales of the PRAQ-R2 showed no significant differences. Thus, apart from a decrease in the concerns over the health of their babies, all women regardless of trimester seem to be occupied with the same matters throughout all trimesters. Significant differences could also be found between nulliparous and multiparous women for the subscales Childbirth Concerns, Attitudes Towards Childbirth, and Avoidance, with scores being lower in multiparous women. Thus, previous experiences with birth and labour might help to reduce fear [19, 54–57]. However, there might be a selection bias. Only women who had a somewhat satisfactory previous birth experience might have wanted another child [58, 59]. Even though multiparous women seem to be less excited about their current pregnancy, the PrA they experience is lowered. On the contrary, low acceptance of pregnancy correlates positively with PrA in women who have not given birth before.

### Strengths and limitations

This study has several strengths. To our knowledge, the translated version of the PrAS is the second German questionnaire for the assessment of PrA and offers expedient features. Since its subscales assess integral aspects of anxiety during pregnancy, a more comprehensive as well as differentiated depiction of PrA across all trimesters is possible. Even though the PrAS-G consists of 32 items and is comparably longer than the PRAQ-R2, its applicability in a clinical context is recommendable since the PrAS offers a wide range of information on PrA. The differentiation of its scales allows for the identification of individual profiles of PrA in pregnant women. Thus, the PrAS represents a useful diagnostic tool for the assessment PrA for women across all trimesters of pregnancy.

However, there are also some limitations that need to be addressed. The study design was cross-sectional and therefore we cannot provide data on intraindividual changes in PrA over the course of pregnancy. A recent study showed that sum scores of the PRAQ-R2 seem to be relatively stable during pregnancy, but scores of subscales change [60]. Since the PrAS consists of more subscales, studies with longitudinal designs should be conducted to examine trajectories of PrA and its diverse facets as reflected by the PrAS. Besides, our sample included participants with and without mental disorders. Further research on levels of PrA in women with preexisting mental disorders could be particularly relevant, since a higher fear of childbirth in women who suffered from anxiety and depression even before pregnancy has been shown [61]. Furthermore, participants filled in the survey online, which allows for low-threshold participation. In addition, most women were highly educated. Both aspects might have caused a selection bias and future studies should incorporate more diverse samples and use strategies to enhance representability of the sample [62].

Future studies should also include men to further decrease the relative neglect of studies on the peripartum mental health of (expectant) fathers [63, 64]. As several items of the PrAS are ineligible for men (e.g. ‘I worry that I will tear or need to be cut during the birth’, ‘I feel scared that I will never regain my figure), a version for men should be developed. This could provide a more holistic approach to parental peripartum mental health and could prevent (expectant) fathers from experiencing clinically relevant symptoms of anxiety in the long run. This would also prevent children and the entire family from suffering further negative consequences caused by spill-over effects [65, 66].

Furthermore, the study has been conducted during the ongoing COVID-19 pandemic, which might have led to higher scores in the PrAS than before the pandemic as has been shown in previous studies [67–70]. However, the psychometric properties of the PrAS are most likely unaffected by this. Thus, with its factorial structure, convergent and divergent validity with other measures, and the ability to identify differences in symptom scores between subsamples of women, the PrAS is a magnificent tool for the assessment of PrA.

### Conclusions

Taken together, our findings demonstrate that the PRAS-G has sound psychometric properties and is recommendable both for clinical practice and scientific purposes. In relevant analyses, high reliability, the eight-factorial structure, as well as convergent and divergent validity were confirmed. Furthermore, the PrAS-G showed the ability to discern between women who either planned or did not plan their pregnancy, were in varying trimesters, and differed in parity.

This knowledge will improve the possibilities to interpret unique compositions of PrA in individual women. Expectant mothers seem to differ in their experience of PrA and tailored interventions on an individual level as well as in public health campaigns are needed to tackle the most pressing aspects of PrA with regard to influencing factors, such as parity [71]. With this approach adverse effects of PrA can be prevented and will instead improve birth experience and well-being of expectant mothers and fathers [8]. Future research should also examine, if childhood development and mental health of children as well as couple and family relationships could be influenced in a positive manner by developing and applying adequate interventions for PrA [8, 16, 72]. Thus, the PrAS-G will be a useful tool for application in a clinical and research context.

## Data Availability

The dataset used and analysed during the current study is available from the corresponding author on reasonable request.

## Abbreviations

PrAS: Pregnancy-related Anxiety Scale
PRAQ-R2: Pregnancy-Related Anxiety Questionnaire-Revised 2
EPDS: Edinburgh Postnatal Depression Scale
DASS-21: Depression, Anxiety and Stress Scale-21
DASS-Anxiety: subscale Anxiety of the Depression, Anxiety and Stress Scale-21
BRS: Brief Resilience Scale
CFA: Confirmatory factor analysis
RMSEA: Root Mean Square Error of Approximation
SRMR: Standardized Root Mean Square Residual
CFI: Comparative Fit Index
TLI: Tucker-Lewis Index
PrAS G: German version of the Pregnancy-related Anxiety Scale
